# Emergence of Lassa Fever Disease in Northern Togo: Report of Two Cases in Oti District in 2016

**DOI:** 10.1155/2017/8242313

**Published:** 2017-12-17

**Authors:** Akouda Akessiwe Patassi, Dadja Essoya Landoh, Agballa Mebiny-Essoh Tchalla, Wemboo Afiwa Halatoko, Hamadi Assane, Bayaki Saka, Mouchedou Abdoukarim Naba, Issifou Yaya, Kossi Atsissinta Edou, Tsidi Agbeko Tamekloe, Abiba Kere Banla, Kokou Mawule Davi, Magloire Manga, Yao Kassankogno, Dominique Salmon-Ceron

**Affiliations:** ^1^Service des Maladies Infectieuses, CHU Sylvanus Olympio, Lomé, Togo; ^2^Comité national de lutte contre les Urgences, Lomé, Togo; ^3^Organisation Mondiale de la Santé, Lomé, Togo; ^4^Direction Préfectorale de la santé de Sotouboua, Sotouboua, Togo; ^5^Institut National d'Hygiène-Laboratoire, Lomé, Togo; ^6^Direction Préfectorale de la santé de Tchamba, Tchamba, Togo; ^7^Service de Dermatologie CHU Sylvanus Olympio, Lomé, Togo; ^8^Direction Préfectorale de la santé de Doufelgou, Niamtougou, Togo; ^9^Aix Marseille Université, INSERM, IRD, SESSTIM, Sciences Economiques & Sociales de la Santé & Traitement de l'Information Médicale, Marseille, France; ^10^Direction Préfectorale de la santé de Oti, Mango, Togo; ^11^Division de l'Epidémiologie du Ministère de la Santé, Lomé, Togo; ^12^Université de Casamance, Ziguinchor, Senegal; ^13^Service de Maladies Infectieuses, Hôpital Cochin, Paris, France

## Abstract

**Background:**

Lassa fever belongs to the group of potentially fatal hemorrhagic fevers, never reported in Togo. The aim of this paper is to report the first two cases of Lassa fever infection in Togo.

**Case Presentation:**

The two first Lassa fever cases occurred in two expatriate's health professionals working in Togo for more than two years. The symptoms appeared among two health professionals of a clinic located in Oti district in the north of the country. The absence of clinical improvement after antimalarial treatment and the worsening of clinical symptoms led to the medical evacuation. The delayed diagnosis of the first case led to a fatal outcome. The second case recovered under ribavirin treatment.

**Conclusion:**

The emergence of this hemorrhagic fever confirms the existence of Lassa fever virus in Togo. After a period of intensive Ebola virus transmission from 2013 to 2015, this is an additional call for the establishment and enhancement of infection prevention and control measures in the health care setting in West Africa.

## 1. Background

**Figure 1:**
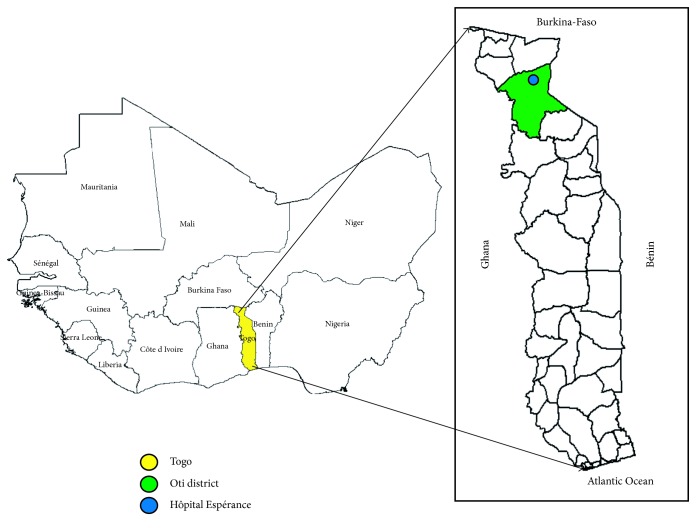
Geographical location of Oti district where “Hôpital de l'Espérance” is located.

Lassa fever is an old hemorrhagic fever discovered for the first time in West Africa in Nigeria in 1969. The single-stranded RNA virus was isolated for the 1st time from the town of Lassa in Nigeria [1–3]. The animal reservoir of the virus is the multimammate mouse (*Mastomys natalensis*). The virus is transmitted to humans via contact of human food with urine or feces of an infected animal living in the house or in the bush [2, 4]. Person-to-person infections and laboratory transmission are also possible, particularly in the absence of adequate infection control measures in the health care environment. Sexual transmission has been described in humans [3]. All humans can develop the disease after infection [1, 3, 4]. The incubation period varies from 3 to 21 days after contact with a Lassa fever patient or contaminated food or water. In 80% of the cases, the infection is asymptomatic or a mild illness with mild fever, malaise, vomiting, or muscle pain. In 20% of the cases, the clinical presentation is severe with externalized bleeding from the mouth or gastrointestinal tract. Digestive, respiratory, and neurological disorders (convulsive seizures, hearing deficit, meningitis, and encephalitis) have been described [1, 3–5]. The reported actual illness-to-infection ratio in endemic areas is presumably 20% [5, 6]. Approximately, 15–20% of patients hospitalized for Lassa fever die from the illness. The case fatality rate may reach 50% in hospitalized patients during occasional Lassa fever epidemics [2, 4]. In 2014, Shaffer et al. reported a case fatality rate of 69% in Sierra Leone [7]. Ribavirin administered early during the first six days of symptoms significantly reduces the fatality rate from 55 to 5%. In endemic areas, 300,000 to 500,000 cases of Lassa fever are estimated to occur yearly, leading to 5,000 to 10,000 deaths in West African countries such as Nigeria, Liberia, Sierra Leone, Guinea, and Mali. Neighboring countries have also declared the presence of the rodent vector of Lassa virus and the occurrence of epidemics in this region [5, 8–12]. Togo, a West African country (Figure 1), had so far reported no Lassa fever cases on its soil. The prediction of the occurrence of Lassa virus in various geographical regions, compatible with the ecology of the rodent reservoir, showed that the emergence of clinical Lassa fever is not unexpected in some West African countries including Togo [12].

In a context of providing health care workers in Togo with a working tool to combat Ebola disease, are the preparedness and alert measures implemented in the Savanes health region enough to detect timely hemorrhagic fever diseases?

The suspicion of recent Lassa fever led to take preventive and infection control measures around the environment of these cases according to WHO guidelines.

## 2. Case Presentation

A 47-year-old man, surgeon in “Hôpital de l'Espérance,” a private hospital in the north of Togo about 550 km from Lomé, the capital [13], consulted in the same hospital on February 12, 2016, for fever. He was vaccinated against yellow fever. The thick smear performed was positive for malaria and received artemether/lumefantrine without any favorable clinical course after six days. Due to the persistence of high-grade fever and a generalized weakness, he was hospitalized in a private room and a nurse was designated for his care. On day 6 after consultation, he complained of fever, chills, malaise, headaches, pharyngitis hypoacusis, and diffuse abdominal pain. Laboratory tests showed leukopenia (1,100 cells/mm^3^) and thrombocytopenia (65,000/mm^3^). His epigastric pain gradually exacerbated. Based on syndromic abdominal pain with febrile illness, an exploratory laparotomy was conducted, which was followed by a bleeding on the operative wound. He was thus air-lifted using an air ambulance from Togo to Cologne, Germany, on day 13. Before the evacuation, the patient was transfused with two units of packaged red blood cells in the airport as his hemoglobin dropped to 6.8 g/dL with evidence of hemolysis symptoms. Despite intensive medical care in Germany, the patient's condition deteriorated rapidly on the second day of admission, and he died on day 14. A postmortem diagnosis of Lassa fever was confirmed by polymerase chain reaction (PCR) in Germany [13].

On March 5, 2016, the 33-year-old health care professional who took care of the surgeon presented with fever, headaches, chills, and body aches. He then had abdominal pain, generalized fatigue and myalgia, and diarrhea. The rest of the clinical status was normal. He was vaccinated against yellow fever. A thick smear was performed, and the patient received antimalarial treatment (artemether/lumefantrine) without success. Laboratory results showed a hemoglobin level of 7 g/d, a white cell count of 3300 × 109/L, and a platelet count of 30 × 109/L. The sedimentation rate was 17 mm at the 1st hour. The patient's renal function was impaired with urea increase to 27.5 mmol/L and creatinine increase to 637 mmol/L. The liver function tests were also impaired with a total bilirubin level of 50 μmol/L and aspartate aminotransferase (AST) and alanine transaminase (ALT) levels of 1321 IU/L and 1060 IU/L, respectively.

Based on the clinical presentation, and the fact that both were health workers in the same hospital, the hypothesis of a viral hemorrhagic fever (Lassa fever) was suggested, and the blood sample of this second patient was taken and sent to Accra at Noguchi Memorial Institute for Medical Research (Ghana) and at “Centre National de Recherche des Fièvres hémorragiques de l'Institut Pasteur” of Lyon (France). A reverse transcriptase polymerase chain reaction (RT-PCR) assay was performed on the blood sample on day 5 after onset, which confirmed the presence of Lassa fever virus. The same day, intravenous (IV) ribavirin regimen was initiated with a loading dose of 30 mg/kg IV (maximum, 2 g) followed by 17 mg/kg IV (maximum, 1 g/dose) every 6 hours for 4 days, and then 8 mg/kg IV (maximum, 500 mg/dose) every 8 hours for 6 days in addition to other supportive treatment. On day 7, the patient was evacuated to Atlanta aboard an air ambulance. The patient gradually regained his strength and completed 10 days of ribavirin with supportive treatment and appropriate nutrition. He was discharged in May 2016 with hearing disorders. These disorders persisted during the first six months after leaving the hospital.

### 2.1. Contact Tracing

This tracing was done on two axes: (i) the tracing of the so-called primary cases and (ii) the follow-up of the contacts around the two cases described above.

Primary cases were suspected to be the source of the contamination of these two cases. The search of these primary cases was done through the review of the medical records of all patients who consulted in the health facilities of the region from January 1 to April 4, 2016. Death tracing in the villages of the area was performed from December 1, 2015 to March 23, 2016.

Among patients who went through the operating room from January 14 to February 2, 2016, three patients were suspected to be primary cases from a total of 78 patients operated on by the same surgeon.

 
*Case 1*. A 7-month-old child, who resided in Sadori (north Togo, Oti district), presented with a persistent fever and a cervical tumefaction. He was operated on February 8; he presented with bleeding of operative wounds and died 3 days later.
 
*Case 2*. An 8-year-old child residing in Kankandou, Oti district, presented with a fever, swelling of the face, and hematuria.
 
*Case 3*. A 31-year-old woman residing in Gando, Oti district, presented with a suspicion of abortion; but after analyzing her history, the case of abortion due to Lassa fever was rejected.

Blood samples for confirmation were taken from the 8-year-old child. Samples were taken also from the mother of the 7-month-old child because of the close contact between them. The search for anti-Lassa IgM and IgG using an enzyme-linked immunosorbent assay (ELISA) technique was negative for this 8-year-old child residing in Kankandou. For the mother of Case 1, the IgG was positive. These tests were performed at Noguchi Memorial Institute for Medical Research (Ghana). The surgeon was consulted for medical advice in outpatient clinics and hospitalizations. Of the 2,051 files reviewed, 36 cases with febrile diseases were selected. Five (05) of the 36 files were cases with arguments in favor of suspect cases.

In public health centers of the region, 19,074 patients were seen of whom 7319 cases were consulted for fever. None of the 7319 cases met the case definition of Lassa fever. There were 21 cases of spontaneous abortions recorded without any context of fever.

After the occurrence of the two Lassa fever cases, 110 contacts were identified and followed up for 21 days. There were 81 contacts among health workers: 80 from Hôpital de l'Espérance and one ambulance driver from Mango military camp. In the community, 29 contacts were followed up by the nurses in health facilities and community health workers. None of the 110 contacts developed symptoms.

## 3. Discussion

This first Lassa fever outbreak was reported in Togo in a context of the epidemiological transition of malaria in Africa. Indeed, following the large mobilization of funding to support malaria control strategies in low- and middle-income countries, a decrease in morbidity and mortality due to malaria in most of the African countries has been noted since a few years [14, 15]. However, malaria remains a major cause of consultation and hospitalization in sub-Saharan African countries including Togo. Therefore, malaria is the first diagnosis suspected by health workers and community health workers, in case of fever in children, pregnant women, travelers, and adults [14, 16, 17]. In this context, it is difficult to diagnose nonmalarial fevers from the first consultation. The two patients in our study were foreigners, one more reason to think first about malaria. This led to a delayed diagnosis and a worsening of the patient's clinical conditions. It is well known that the delay in the diagnosis of hemorrhagic fevers is the starter of most outbreaks reported, increases the rate of human-to-human transmission, and finally, makes it difficult to stop the dissemination of the disease [16, 18]. It becomes urgent to explain to the patients and convince health workers that there are many causes of fever. Fever is a major symptom of hemorrhagic diseases transmitted (i) from human to human in the hospital, (ii) by diurnal mosquitoes, (iii) by ticks or fleas, and (iv) by animals (domestic and game). Some hemorrhagic fevers are lethal without treatment but less frequent. Fever during malaria is more often severe [16, 19, 20]. Our first reported case began with fever. The positive result of the thick smear and the duration of malaria treatment by artemether were the factors that delayed the true diagnosis of Lassa fever and led to a fatal outcome.

### 3.1. Prevention and Response Measures

Once the hypothesis of Lassa fever was established, measures were taken to limit the dissemination of the virus and regain the eventual sources of contamination within the hospital. This outbreak of Lassa fever occurred in a dual context of Ebola disease in the West African subregion and of a Lassa fever outbreak in Benin and Nigeria. Nigeria experienced an Ebola epidemic in 2014 and a reemergence of Lassa fever in 2016. The epidemic of Lassa fever reappeared in Benin in 2015 and 2016. This is evident that arenaviruses are reemerging in African countries due to climatic or ecological changes and sometimes resulting in case fatalities.

The primary transmission occurs from the natural reservoir to the human. Once the first case appears, the transmission becomes human to human and the family or the health care workers are at risk. This explains the occurrence of the second case of nosocomial Lassa fever in Oti district. This second case was a health care professional who took care of the first Lassa fever case. At the onset of symptoms, the patient was isolated at home. The good level of hygiene in this hospital has limited the transmission although this first fatal case was operated upon in the same hospital. The other factor at risk of expansion of the infection was the long delay for diagnostic confirmation, a delay of 3 weeks which was too long due to the need to send the samples for diagnosis confirmation in Ghana and France [4]. This shows the importance of having locally the means of rapid diagnosis and qualified person for hemorrhagic fever diagnosis. The diagnosis of the first case was post mortem, but the country started preparing since 2014 for the fight against Ebola declared in Guinea while human cases of Lassa fever were already present in the neighboring countries Benin and Nigeria. Moreover, despite these risks, the country did not have ribavirin in any public or private health centres. Fortunately, the second case has received an intravenous ribavirin regimen.

Several measures have been rapidly implemented locally to stop the transmission: sorting of patients in the regional hospitals of the region on the basis of their clinical signs and country of origin (Benin, Nigeria, and Burkina Faso). Hospital hygiene measures were also enhanced (implementation of handwashing devices; decontamination and disinfection of medical equipment after use; provision of personal protective equipment; sorting, collection, storage, and destruction of biomedical waste; disinfection of the surgeon's home (index case); installation of a tent at Hôpital de l'Espérance, to serve as an isolation room for suspected cases; follow-up of contact cases by recording body temperature twice daily; and briefing of laboratory staff on sampling, processing, and infection control measures).

Hôpital de l'Espérance opened its doors in 2015 by an American Baptist mission. It has a medical service, a general surgery ward, an obstetric gynecology ward, a general laboratory, and an administrative block. More than a dozen African nationalities seek health care in this hospital.

Public sensitization was conducted through communication and social mobilization in public places and the production of health messages on the media. The Emergency Management Committee Meeting conducted training for health personnel of the district on Lassa fever, and urgent measures were taken for prevention and control of the disease. A district response plan and supervision of health sites plan on the implementation of guidelines for prevention and control measures were developed. Radio programs translated into local languages on the two radio channels of the district were broadcast. Sensitization sessions were organized in schools, mosques, churches, and at the border (Gando).

## 4. Conclusion

Togo experienced its first Lassa fever outbreak in the health district of Oti, North Togo. The source of the infection of the first case remained inconclusive: the serological research in the indigenous population and the animal reservoir remain a determinant element to guide preventive measures. Health workers in the region should keep in mind the existence of Lassa fever infection in the differential diagnosis of various fevers during consultations.

## Additional Points

The data sets supporting the conclusions of this article are included within the manuscript and its supporting material.
